# Association Between Plasma ADAMTS-7 Levels and Diastolic Dysfunction in Patients with Type 2 Diabetes Mellitus

**DOI:** 10.3390/medicina60121981

**Published:** 2024-12-02

**Authors:** Asimina Ganotopoulou, Emmanouil Korakas, Loukia Pliouta, Aikaterini Kountouri, Sotirios Pililis, Stamatios Lampsas, Ignatios Ikonomidis, Loukianos S. Rallidis, Athanasia Papazafiropoulou, Andreas Melidonis, Vaia Lambadiari

**Affiliations:** 1 Research Unit and Diabetes Centre, 2nd Department of Internal Medicine, Attikon Hospital, Medical School, National and Kapodistrian University of Athens, 124 62 Athens, Greece; asimigio4@gmail.com (A.G.); mankor-th@hotmail.com (E.K.); plioutaloukia@gmail.com (L.P.); katerinak90@hotmail.com (A.K.); sotiris181@yahoo.gr (S.P.); 2 Department of Internal Medicine and Diabetes Centre, Tzaneio General Hospital of Piraeus, 185 36 Piraeus, Greece; athpapazafiropoulou@gmail.com (A.P.); melidonisa@yahoo.com (A.M.); 3 2nd Department of Ophthalmology, Attikon Hospital, Medical School, National and Kapodistrian University of Athens, 124 62 Athens, Greece; lampsas.stam@gmail.com; 4 2nd Department of Cardiology, Attikon Hospital, Medical School, National and Kapodistrian University of Athens, 124 62 Athens, Greece; ignoik@gmail.com (I.I.); lrallidis@gmail.com (L.S.R.)

**Keywords:** biomarkers, diabetes mellitus, heart failure, ADAMTS-7, metalloproteinases, atherosclerosis, diastolic dysfunction

## Abstract

A disintegrin and metalloproteinase with thrombospondin motifs-7 (ADAMTS-7) belongs to the family of metalloproteinases that contributes to tissue homeostasis during morphogenesis and reproduction. These metalloproteinases regulate various cell functions such as cell proliferation, are important regulators in tissue regeneration, and play a role in vascular remodelling, which is involved in atherosclerosis development. Despite the well-established association between ADAMTS-7 and atherosclerotic disease, data regarding the metalloproteinase’s association with LV function remain scarce. The aim of this study was to investigate the association of ADAMTS-7 levels with diastolic dysfunction and various echocardiographic parameters in patients with type 2 diabetes mellitus. All patients underwent a clinical, vascular, and echocardiographic examination during their visit. Plasma ADAMTS-7 levels were measured in all patients. The results showed that diastolic dysfunction was strongly associated with age, but had no statistically significant association with ADAMTS-7. When individual echocardiographic parameters were examined, ADAMTS-7 levels showed a positive tendency only with deceleration time (DT), with the other echocardiographic parameters being positively associated only with age. The possible role of ADAMTS-7 in diastolic dysfunction and in the development and progression of heart failure in patients with type 2 diabetes mellitus deserves further investigation.

## 1. Introduction

Heart failure (HF), either with reduced (HFrEF), mildly reduced (HFmrEF), or preserved ejection fraction (HFpEF), is an ever-growing public health problem worldwide, affecting over 23 million patients [1]. Approximately half of the patients who receive a diagnosis of HF in the USA annually have diastolic dysfunction. In the EPICA study in Portugal, which enrolled 5434 patients attending primary care centres in 1998, the overall prevalence of HF was 4.4%, and the prevalence of diastolic dysfunction was 1.4%; major risk factors included age, arterial hypertension, and diabetes mellitus. Specifically, in patients with diabetes, diastolic dysfunction is associated with increased left ventricular (LV) mass, wall thickness, and arterial stiffness, which in turn is a well-established marker of subclinical atherosclerosis and chronic inflammation in this patient population [2]. Of note, 34% of patients with diabetes have diastolic dysfunction [2]. Many different mechanisms have been proposed over the years, including alterations in intracellular Ca^2+^ transients, increased reactive oxygen species (ROS) and oxidative stress, increased advanced glycation end-products (AGEs), mitochondrial dysfunction, and reactive interstitial fibrosis [3].

Ventricular remodelling plays an essential role in the development of heart failure, and the structure of the left ventricle is largely dependent on the properties and integrity of the extracellular matrix (ECM) [3]. The activation of extracellular proteases promotes ECM degradation, and this process further promotes ventricular remodelling. In this pathophysiological pathway, matrix metalloproteinases (MMP) play a crucial role [4]. A disintegrin and metalloproteinase with thrombospondin motifs (ADAMTS) is a family of 19 peptidases that contribute to the structural changes in the ECM in a way that largely mimics other MMPs, but with higher specificity [5]. ADAMTS-7, a member of this family, was first identified in 1999 [6]. For years, the physiological functions of ADAMTS-7 were mainly associated with its involvement in the pathogenesis of arthritis, as its main substrate, cartilage oligomeric matrix proteins (COMP), is found in the extracellular matrix of cartilage. However, two genome-wide association studies (GWAS) [7,8] identified the ADAMTS-7 gene as a novel locus for the development of coronary atherosclerosis. Wangetal [9] showed that ADAMTS-7 contributed to vascular remodelling by mediating vascular smooth muscle cell migration and neointima formation in animal models. Regarding cardiac function, Wu et al. [10] showed that ADAMTS-7 levels were positively correlated with markers of cardiac dysfunction after acute myocardial infarction, and that ADAMTS-7 serum levels were lower in patients with left ventricular reverse remodelling (LVRR) compared to those without LVRR [11]. Many different markers have been employed to assess diastolic dysfunction. For example, deceleration time (DT) is the rate at which the atrial and ventricular pressures equilibrate after onset of the E wave and is shorter in compliant ventricles (160–240 ms in adults). In the early stages of diastolic dysfunction, the decreased LV compliance leads to increased LV filling pressure, delaying emptying and thus prolonging the E wave deceleration time (>240 ms) [12]. However, despite the well-established association between ADAMTS-7 and atherosclerotic disease, data regarding the association with LV function remain scarce. In this study, we aimed to investigate the association of ADAMTS-7 levels with diastolic dysfunction and various echocardiographic parameters in patients with type 2 diabetes mellitus.

## 2. Materials and Methods

### 2.1. Patients

This was a single-centre prospective observational study that included patients with type 2 diabetes mellitus (T2DM) from the Diabetes Outpatient Clinic of Tzaneion Hospital. All patients underwent a clinical, vascular, and echocardiographic examination during their visit. Patients were divided into 2 groups: patients with T2DM and diastolic dysfunction, and patients with T2DM without diastolic dysfunction. Exclusion criteria included established heart failure of class NYHA > I, reduced ejection fraction (EF ≤ 50%), history of osteoarthritis, history of thrombotic thrombopenic purpura (TTP), acute or chronic inflammatory states, hepatic dysfunction, active malignancy, immunomodulatory or immunosuppressive medication, and pregnancy, which are all conditions related to alterations in ADAMTS-7 and other metalloproteinases

The study protocol was approved by the ethics committee of Tzaneion Hospital before the beginning of the enrollment and any other procedure. All participants provided written informed consent before study initiation. This study was carried out in accordance with the Declaration of Helsinki.

### 2.2. Sample Collection

Twenty-milliliter samples of whole blood were collected in EDTA anticoagulant tubes and were stored at 4 °C. Samples were centrifuged for 15 min at 1000× *g* at 4 °C within 30 min of collection. Samples were stored at −80 °C until analysis.

### 2.3. Plasma ADAMTS-7 Levels

Plasma ADAMTS-7 levels were measured using an enzyme-linked immunosorbent assay (ELISA) for human ADAMTS-7 (Human ADAMTS-7 ELISA Kit; MyBioSource Inc., San Diego, CA, USA). These assays were performed by an investigator blinded to the sources of the samples.

### 2.4. Echocardiography

Examinations were conducted utilizing a Vivid E95 ultrasound system from GE Medical Systems in Horten, Norway. The digital storage of the studies was carried out on a computerized station (EchoPac GE202, Horten, Norway). Two observers, who were blinded to clinical and laboratory data, independently analysed all of the studies.

### 2.5. Statistical Analysis

Statistical analysis was performed with SPSS statistical package 23.0 (SPSS 23.0, Chicago, IL, USA). Continuous variables were tested using the Kolmogorov–Smirnov test to assess the normality of the distributions. For continuous variables, the differences between two groups were examined using an independent samples *t*-test. For continuous variables without a normal distribution, the differences between two groups were examined using Mann–Whitney test. For categorical clinical variables, differences between groups were evaluated using chi-square tests. Pearson’s correlation coefficient (r) was used to examine the associations between quantitative variables. Multiple logistic regression analysis was used to investigate independent associations between echocardiographic indices and the variables studied. A 2-sided *p* < 0.05 was considered significant.

## 3. Results

### 3.1. Demographic and Anthropometric Characteristics

**Figure 1:**
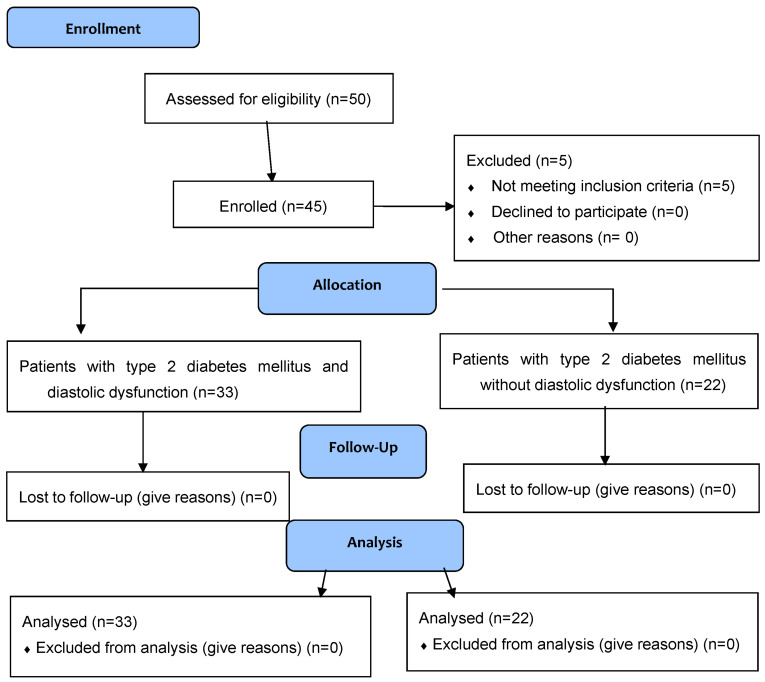
CONSORT flow diagram.

Overall, 45 consecutive patients (46.7% men) were enrolled (Figure 1). The mean age was 56.3 ± 10.6 years, the mean body mass index (ΒΜΙ) was 30.7 ± 3.6 Kg/m^2^, and the mean glycosylated hemoglobin (HbA1c) was 7.1 ± 0.6%. A total of 35 patients (77.8%) had a history of arterial hypertension and 26 patients (57.8%) had dyslipidemia. All patients received metformin as a first-line antidiabetic treatment, and 17 patients (37.8%) received DPP-4 inhibitors (DPP-4is), 13 patients (28.9%) received GLP-1 receptor agonists (GLP-1RAs), 5 patients (11.1%) received SGLT-2 inhibitors (SGLT-2is), 8 patients (17.8%) received sulfonylureas, and 9 patients (20%) received insulin as an add-on medication to metformin (Table 1 and Table 2).

### 3.2. Diastolic Dysfunction

Logistic regression analysis showed a positive correlation only between diastolic function and age (*p* < 0.001) (Table 3). No statistically significant association between diastolic dysfunction and ADAMTS-7 levels was shown. Gender-based analysis did not show any significant association between diastolic dysfunction and the examined variables. 

### 3.3. Left Atrial Volume Index (LAVI)

Logistic regression analysis showed a positive correlation only between LAVI and age (*p* = 0.05) (Table 4). No statistically significant association between LAVI and ADAMTS-7 levels was shown.

### 3.4. Deceleration Time (DT)

Linear analysis showed a marginally nonsignificant positive association between DT index and ADAMTS-7 levels (*p* = 0.06) (Table 5). 

### 3.5. E/E’ Ratio

Logistic regression analysis showed a positive correlation only between E/E’ ratio and age (*p* = 0.03) (Table 6). No statistically significant association between E/E’ ratio and ADAMTS-7 levels or other variables was shown.

## 4. Discussion

The aim of the present study was to examine the association between ADAMTS-7 levels and diastolic dysfunction in patients with T2DM. The results showed that diastolic dysfunction was strongly associated with age, but had no statistically significant association with ADAMTS-7. When individual echocardiographic parameters were examined, ADAMTS-7 levels showed a positive tendency only with deceleration time (DT), with the other echocardiographic parameters being positively associated only with age. 

The absence of any significant association between ADAMTS-7 levels and diastolic dysfunction can be considered surprising, taking into account that the role of ADAMTS-7 in cardiovascular disease has been established in both preclinical and clinical models. In fact, however, the vast majority of data refer to the role of ADAMTS-7 as a potent risk factor for atherosclerosis, and not cardiac function. In three recent GWAS studies, the variants rs4380028, rs1994016, and rs3825807 in the ADAMTS-7 gene were associated with coronary artery disease (CAD) and, even more specifically, the rs3825807 G/G genotype had a negative association with atherosclerosis prevalence and severity [7,8]. In apolipoprotein E (Apoe) knockout (Apoe−/−) and LDL receptor knockout (LdLr−/−) mice, which both have a hyperlipidemic phenotype, the deletion of the ADAMTS-7 gene significantly reduced atherosclerotic lesion formation in the aorta and aortic root, without significant changes in blood lipid levels [13]. Apart from animal models, human studies have also demonstrated similar results. Yu et al. [14] recruited 182 CAD patients who were divided into subgroups based on ADAMTS-7 levels and their Syntax score. It was shown that plasma ADAMTS-7 levels in the high-Syntax-score group were significantly higher than those in the low-Syntax-score group, and they also significantly and positively correlated with the Syntax score tertiles (r = 0.157, *p* = 0.035), although no difference was noted for event-free survival rate. This was the first large-scale human study to translate the previous laboratory findings back to the clinic, and it rendered ADAMTS-7 as a possible factor that may be involved in the severity of coronary artery disease. Similar results were shown by Sharifi et al. [15], who reported that ADAMTS-7 expression was higher in unstable carotid plaques as compared to stable carotid plaques in humans, and by Bengtsson et al. [16], who showed that carotid plaques from symptomatic patients showed increased levels of ADAMTS-7 compared with lesions from asymptomatic patients. In a recent meta-analysis [17], the ADAMTS7 polymorphism was confirmed to be an important risk factor not only for the development of CAD but also for CAD progression in already existing lesions.

On the other hand, data regarding the effect of ADAMTS-7 levels specifically on the myocardium and, therefore, cardiac function, are scarce. Our study can only be compared to two large-scale studies where LV function was investigated. In the first study, Wu et al. [10] compared patients with acute myocardial infarction (AMI) to controls, and patients were stratified according to a left ventricular ejection fraction (LVEF) of ≤35% or >35%. It was shown that plasma ADAMTS-7 levels were higher in patients with a LVEF of ≤35% compared with those with an LVEF of >35% (6.73 ± 2.47 vs. 3.22 ± 2.05 ng/mL, *p* < 0.05), and that they were positively correlated with left ventricular mass index (LVMI), left ventricular end-diastolic diameter (LVEDD), and left ventricular end-systolic diameter (LVESD). In the same way, in another work by this group in patients after an ST-elevation myocardial infarction (STEMI) [11], serum levels of ADAMTS-7 in patients with left ventricular reverse remodelling (LVRR) were lower than in those without LVRR (3.84 ± 2.26 vs. 5.02 ± 2.54, *p* = 0.032) seven days after STEMI, and ADAMTS-7 levels were indicated as an independent predictor of LVRR. It must be noted, however, that in our cohort, subjects did not have any history of coronary artery disease, neither chronic nor acute, and thus, a direct comparison would be rather audacious, both from a clinical and methodological perspective. In addition, in our study, we focused on diastolic dysfunction, which meant that all our patients had a preserved ejection fraction. Even more importantly, all our patients suffered from T2DM and received appropriate medication; the possible effects of antidiabetic agents on ADAMTS-7 levels cannot be ignored. An important detail in the study by Yu et al. [14] was that ADAMTS-7 levels correlated with a history of hyperlipidemia, but not with the lipid levels themselves. This implied that ADAMTS-7 possibly interacts with the traditional risk factors in a rather chronic way, but it also further supported the notion that ADAMTS-7 levels and activity are more closely associated with chronic inflammation. In vitro findings showed upregulation of ADAMTS7 expression in vascular smooth muscle cells (VSMCs) by inflammatory cytokines such as TNF-a, but not by anti-inflammatory cytokines (TGFB) or oxidized LDL (ox-LDL) [6]. Therefore, the well-established anti-inflammatory properties of antidiabetic medications, especially GLP-1RAs and SGLT-2is [18], may have played a role in the associations shown in our cohort, although the small sample size does not allow for robust conclusions to be drawn.

Apart from clinical factors, the reasons behind the discrepancy between our results and the existing literature also need to be sought regarding the pathophysiological mechanisms through which ADAMTS-7 exerts its actions. Diastolic dysfunction is the cardinal trait of diabetic cardiomyopathy, a type of diastolic heart failure whose pathogenesis has not yet been fully elucidated. Regardless of the cause, myocytes and their surrounding extracellular matrix (ECM) respond to injury by reorganizing, leading to progressive ventricular dilatation, wall thinning, and cardiac dysfunction [19]. In the failing heart, the myocardial matrix is degraded by matrix metalloproteinases (MMPs) as a result of the imbalance between them and their natural inhibitors, the tissue inhibitors of metalloproteinases (TIMPs), which include four distinct species (TIMP-1, -2, -3, and -4) [20]. ADAMTS-7 degrades ECM by degrading cartilage oligomeric matrix protein (COMP). Huang et al. [21] reported the presence of COMP in cardiomyocytes, thus proposing a possible role for ADAMTS-7 in the initiation and progression of dilated cardiomyopathy. The degradation of COMP in cardiac fibroblasts specifically diminishes the production of type I collagen, which in turn disrupts the structure of ECM and promotes ventricular dilatation [22]. However, the exact pathways through which ADAMTS-7 affects cardiac remodelling are poorly known. Despite the fact that ADAMTS-7 is widely expressed in the heart and large vessels and mediates vascular smooth muscle cell migration, knockout of ADAMTS-7 under physiological conditions has no significant effect on the cardiac structure and function in mice, despite reducing atherosclerosis at the same time [13]. In a rat model, ADAMTS-7 was poorly expressed in myocardial cells 28 days after myocardial infarction [23]. In addition, other various MMPs, such as MMP-2, participate in left ventricular remodelling, which renders the individual contribution of ADAMTS-7 quite obscure [24]. Finally, the levels of ADAMTS-7 per se may not be of utmost importance for the clinical implications of the enzyme. In one of the aforementioned GWAS studies, the rs3825807 G/G genotype, which is associated with lower atherosclerosis prevalence and severity, does not alter the expression and, thus, the levels of ADAMTS7, but rather hinders its activity, resulting in reduced COMP degradation [25]. In a recent work by Tastemur et al. [26], the levels of ADAMTS-7 did not differ between diabetic and prediabetic subjects and controls, despite the chronic inflammation and oxidative stress that insulin resistance entails. In general, the exact manner in which ADAMTS-7 eventually affects the cardiac muscle is still vague, and as more substrates and genetic polymorphisms are discovered, the quantitative and qualitative properties of this enzyme are going to be more clearly translated into clinical applications.

Our study is the first one to investigate the role of ADAMTS-7 in diastolic dysfunction in asymptomatic patients with type 2 diabetes mellitus. Among the limitations of the study is its observational nature, which does not allow for robust causal relationships to be established. Secondly, it is a single-centre study with a small sample size. In addition, ADAMTS-7 was only tested in the peripheral blood and not in the myocardium. Nevertheless, it adds some useful insights to the existing literature, as the role of ADAMTS-7 in cardiac function has been investigated only in a few studies. More large-scale studies are necessary to further evaluate the potential mechanisms involving the role of ADAMTS-7 in heart failure.

## 5. Conclusions

ADAMTS-7 levels are not associated with the presence of diastolic dysfunction in patients with type 2 diabetes mellitus. When individual indices of cardiac function were studied, only a marginally nonsignificant positive association between DT index and ADAMTS-7 levels was observed. Prospective studies with a larger sample size are necessary to assess the possible role of ADAMTS-7 in the development and progression of heart failure.

## Figures and Tables

**Table 1:** Characteristics of the study population by gender. HbA_1c_: glycosylated hemoglobin; DPP-4i: dipeptidyl peptidase 4 inhibitor; GLP-1RA: glucagon-like peptide-1 receptor agonist; SGLT-2i: sodium glucose cotransporter-2 inhibitor; HDL: high-density lipoprotein; LDL: low-density lipoprotein; GFR: glomerular filtration rate; DT: deceleration time; E/E’: early mitral inflow velocity/mitral annular early diastolic velocity ratio; LAVI: left atrial volume index.

**Variable**	**Patients**	**Men**	**Women**	***p* Value**
	-	21 (46.7)	24 (53.3)	-
Age (years)	56.3 ± 10.6	56.8 ± 9.1	55.8 ± 12.0	0.75
Body mass index (kg/m^2^)	30.7 ± 3.6	31.9 ± 3.7	29.6 ± 3.2	0.03
ADAMTS-7 (pg/mL)	0.32 ± 0.1	0.31 ± 0.011	0.33 ± 0.10	0.55
Total cholesterol (mg/dL)	203.1 ± 29.4	204.2 ± 31.9	202.0 ± 27.7	0.81
HDL cholesterol (mg/dL)	45.6 ± 6.1	46.7 ± 6.1	44.8 ± 5.9	0.29
LDL cholesterol (mg/dL)	101.1 ± 22.1	100.9 ± 23.0	101.3 ± 21.8	0.96
Triglycerides (mg/dL)	141.9 ± 25.2	138.8 ± 23.9	144.8 ± 26.9	0.43
GFR (mL/min/1.73 m^2^)	85.5 ± 17.2	81.0 ± 18.4	89.2 ± 15.4	0.11
HbA1c (%)	7.1 ± 0.6	7.3 ± 0.5	6.9 ± 0.6	0.05
Arterial hypertension (%)	35 (77.8)	18 (85.7)	17 (70.8)	0.20
Dyslipidemia (%)	26 (57.8)	12 (57.1)	14 (58.3)	0.58
Diastolic dysfunction (%)	29 (69.0)	15 (78.9)	14 (60.9)	0.12
DT	205.3 ± 56.8	196.8 ± 59.3	212.4 ± 11.2	0.37
E/E’	8.7 ± 3.2	8.4 ± 2.6	8.9 ± 3.6	0.75
LAVI	59.3 ± 10.2	55.2 ± 20.9	63.2 ± 19.2	0.21
**Antidiabetic Medication**	**Patients**	**Men**	**Women**	***p* Value**
Metformin (%)	45 (100)	21 (100)	24 (100)	-
DPP-4i (%)	17 (37.8)	9 (42.9)	8 (33.3)	0.36
GLP-1RA (%)	13 (28.9)	6 (28.6)	7 (29.2)	0.61
Sulfonylurea	8 (17.8)	4 (19)	4 (16.7)	0.57
SGLT-2i (%)	5 (11.1)	3 (14.3)	2 (8.3)	0.44
Insulin (%)	9 (20)	5 (23.8)	4 (16.7)	0.41

**Table 2:** Characteristics of the study population by the presence of diastolic dysfunction. HbA1c: glycosylated hemoglobin; DPP-4i: dipeptidyl peptidase 4 inhibitor; GLP-1RA: glucagon-like peptide-1 receptor agonist; SGLT-2i: sodium glucose cotransporter-2 inhibitor; HDL: high-density lipoprotein; LDL: low-density lipoprotein; GFR: glomerular filtration rate; DT: deceleration time; E/E’: early mitral inflow velocity/mitral annular early diastolic velocity ratio; LAVI: left atrial volume index.

**Variable**	**Patients**	**Diastolic Dysfunction (−)**	**Diastolic Dysfunction (+)**	***p* Value**
	-	33	17	-
Age (years)	56.3 ± 10.6	47.2 ± 10.6	58.6 ± 10.9	0.001
Body mass index (kg/m^2^)	30.7 ± 3.6	30.9 ± 3.5	29.8 ± 3.4	0.32
ADAMTS-7 (pg/mL)	0.32 ± 0.1	0.28 ± 0.10	0.34 ± 0.11	0.08
Total cholesterol (mg/dL)	203.1 ± 29.4	193.6 ± 35.8	208.5 ± 18.5	0.05
HDL cholesterol (mg/dL)	45.6 ± 6.1	46.4 ± 8.4	46.7 ± 5.1	0.88
LDL cholesterol (mg/dL)	101.1 ± 22.1	104.8 ± 24.1	100.9 ± 19.8	0.55
Triglycerides (mg/dL)	141.9 ± 25.2	131.2 ± 37.3	140.8 ± 24.6	0.28
GFR (mL/min/1.73 m^2^)	85.5 ± 17.2	90.9 ± 20.1	86.2 ± 19.8	0.42
HbA1c (%)	7.1 ± 0.6	6.6 ± 1.1	6.9 ± 0.8	0.33
Arterial hypertension (%)	35 (77.8)	8 (47.1)	26 (78.8)	0.02
Dyslipidemia (%)	26 (57.8)	8 (47.1)	16 (48.5)	0.92
DT	205.3 ± 56.8	213.1 ± 34.1	199.9 ± 62.9	0.43
E/E’	8.7 ± 3.2	7.6 ± 4.6	8.8 ± 2.1	0.45
LAVI	59.3 ± 10.2	49.7 ± 13.8	63.5 ± 17.2	0.01
**Antidiabetic Medication**	**Patients**			***p* Value**
Metformin (%)	45 (100)	13 (76.5)	29 (87.9)	0.29
DPP-4i (%)	17 (37.8)	4 (23.5)	13 (39.4)	0.360.26
GLP-1RA (%)	13 (28.9)	6 (35.3)	6 (18.2)	0.610.18
Sulfonylurea	8 (17.8)	3 (17.6)	4 (12.1)	0.570.59
SGLT-2i (%)	5 (11.1)	-	5 (15.2)	-
Insulin (%)	9 (20)	3 (17.6)	5 (15.2)	0.82

**Table 3:** Logistic regression analysis in the study population with diastolic function as the dependent variable. HbA1c: glycosylated hemoglobin; GFR: glomerular filtration rate; BMI: body mass index.

	Τιμή Ρ	Exp(B)	Lower	Upper
Gender (male-to-female)	0.14	4.0	0.63	25.69
Age	0.01	1.2	1.05	1.45
BMI	0.55	0.9	0.67	1.23
GFR	0.37	1.0	0.96	1.10
Arterial hypertension	0.49	0.3	0.02	7.19
Dyslipidemia	0.59	0.6	0.09	3.84
HbA1c	0.28	2.6	0.45	15.88
ADAMTS-7	0.28	166.2	0.02	1,622,244.58

**Table 4:** Logistic regression analysis in the study population with LAVI as the dependent variable. HbA1c: glycosylated hemoglobin; GFR: glomerular filtration rate; BMI: body mass index.

	Beta	*p* Value
Gender (male-to-female)	−0.23	0.18
Age	0.49	0.05
BMI	−0.002	0.99
GFR	−0.12	0.51
Arterial hypertension	−0.12	0.61
Dyslipidemia	0.07	0.66
HbA1c	−0.05	0.72
ADAMTS-7	0.05	0.73

**Table 5:** Logistic regression analysis in the study population with DT as the dependent variable. HbA1c: glycosylated hemoglobin; GFR: glomerular filtration rate; BMI: body mass index.

	Beta	Τιμή Ρ
Gender (male-to-female)	−0.12	0.49
Age	−0.24	0.28
BMI	−0.11	0.56
GFR	0.15	0.38
Arterial hypertension	0.12	0.61
Dyslipidemia	0.25	0.10
HbA1c	0.19	0.22
ADAMTS-7	−0.30	0.06

**Table 6:** Logistic regression analysis in the study population with E/E’ ratio as the dependent variable. HbA1c: glycosylated hemoglobin; GFR: glomerular filtration rate; BMI: body mass index.

	Beta	Τιμή Ρ
Gender (male-to-female)	0.06	0.80
Age	1.06	0.03
BMI	−0.05	0.88
GFR	−0.08	0.74
Arterial hypertension	−0.62	0.14
Dyslipidemia	−0.04	0.85
HbA1c	0.06	0.80
ADAMTS-7	−0.28	0.35

## Data Availability

The data presented in this study are available on request from the corresponding author.
